# Factors Associated with Mental Health Outcomes Following Drug Overdose Loss

**DOI:** 10.3390/bs16091651

**Published:** 2026-09-15

**Authors:** Karli R. Hochstatter, Kalie Quon-Adams, Jamison S. Bottomley

**Affiliations:** 1 Friends Research Institute, Baltimore, MD 21201, USA; 2 Department of Psychiatry and Behavioral Sciences, Medical University of South Carolina, Charleston, SC 29425, USA

**Keywords:** overdose bereavement, drug-related death, mental health, prolonged grief, depression, anxiety, posttraumatic stress

## Abstract

The ongoing overdose crisis has created a widespread but understudied population of bereaved individuals at elevated risk for adverse mental health outcomes. This study examines cognitive, emotional, and interpersonal factors associated with symptom severity of prolonged grief disorder (PGD), major depressive disorder (MDD), generalized anxiety disorder (GAD), and posttraumatic stress disorder (PTSD) among 115 adults who lost a loved one to drug overdose. Participants completed an online survey assessing continuing bonds, centrality of the death event, integration of the loss, unfinished business, resilience, and social support. Multivariable linear regression was used to estimate associations between these factors and severity of mental health outcomes, adjusting for relevant covariates. Results indicated that greater centrality of the death event was consistently associated with higher PGD, MDD, and GAD symptoms, while unfinished business, particularly surrounding unfulfilled wishes, was associated with higher MDD and PTSD symptoms. Continuing bonds were also linked to higher PGD and GAD symptoms. In contrast, greater integration of the loss and higher resilience were associated with lower symptom severity across multiple outcomes, and greater social support was associated with lower depressive symptoms. Findings highlight the multidimensional nature of overdose bereavement and identify potentially modifiable factors that warrant further investigation in longitudinal and intervention research.

## 1. Introduction

### 1.1. Overdose Bereavement

The ongoing drug overdose crisis represents a profound and far-reaching public health challenge in the United States and globally. Over 40% of Americans personally know someone who died from an overdose, underscoring the widespread social impact of substance-related mortality (Harris, 2024). The consequences of overdose extend well beyond the individual, affecting families, social networks, and entire communities. These losses often disrupt lives and can have lasting psychological, social, and health effects.

Increasingly, the overdose crisis is recognized as a form of multigenerational trauma. The rate of children experiencing parental loss to an overdose rose from 27.0 per 100,000 in 2011 to 63.1 per 100,000 in 2021, with a disproportionate impact on minority communities like Black and Native populations (Jones et al., 2024). These patterns highlight that overdose bereavement is not a rare or isolated experience, but rather a widespread phenomenon with significant implications for population health.

### 1.2. Mental Health Outcomes Following Overdose Loss

Given the scale and persistence of overdose-related deaths, understanding the experiences of those bereaved by these losses is critical. Prior research demonstrates that individuals who experience a sudden or unexpected loss are at elevated risk for adverse mental health outcomes and impairment compared to those who experience anticipated deaths (Krychiw et al., 2018). Importantly, this risk appears to be amplified in the context of overdose loss (Bottomley et al., 2022).

Prior research indicates that individuals bereaved by overdose exhibit high rates of psychiatric symptoms across multiple domains. In one study, nearly three-quarters of participants who suddenly lost someone to overdose met the study’s criteria for prolonged grief disorder (PGD), while approximately half reported symptoms consistent with post-traumatic stress disorder (PTSD) and major depressive disorder (MDD), and over one-third reported symptoms consistent with generalized anxiety disorder (GAD) (Bottomley et al., 2022). Notably, these rates were comparable to those observed among individuals bereaved by suicide and substantially higher than those observed following other types of sudden loss. Interpretation of the PGD findings, however, was based on earlier International Classification of Diseases 11th Revision (ICD-11) guidelines that defined PGD using a bereavement threshold of six months. More recently (in 2022), PGD was added to the Diagnostic and Statistical Manual of Mental Disorders, Fifth Edition, Text Revision (DSM-5-TR) and requires symptom persistence for at least 12 months after the death, and few studies to date have examined PGD among overdose-bereaved individuals using the 12-month cutoff. Thus, further research characterizing prolonged grief symptoms among overdose-bereaved individuals within the context of contemporary diagnostic frameworks is warranted. Collectively, existing evidence indicates that overdose bereavement is associated with a substantial burden of psychological distress across multiple symptom domains.

### 1.3. Factors Associated with Bereavement Outcomes

A growing body of literature has begun to identify factors associated with the development and severity of mental health outcomes following sudden loss. Exposure-related characteristics, such as discovering the body, have been associated with increased traumatic stress (Bottomley et al., 2022). Similarly, the degree of shock surrounding the death has been linked to greater psychological distress, including PTSD symptoms and broader mental health difficulties (Feigelman et al., 2023).

Interpersonal and emotional processes also appear to play a critical role. Individuals bereaved by overdose frequently report elevated levels of stigma, guilt, and shame (Bottomley et al., 2025b) which have been shown to contribute to worse mental health outcomes (Bottomley et al., 2025a). These experiences may operate through maladaptive coping processes, such as avoidance, which in turn exacerbate symptoms of prolonged grief and posttraumatic stress (Bottomley et al., 2025a). Additionally, characteristics of the pre-death relationship, including closeness and conflict, often shape post-loss adjustment (Hewson et al., 2024).

Despite these advances, research to date has been relatively fragmented, often focusing on isolated predictors of negative psychological sequelae. As a result, there remains a limited understanding of how cognitive, emotional, and contextual factors impact mental health outcomes following overdose bereavement.

### 1.4. Cognitive and Emotional Processes in Bereavement

To better understand the potential processes associated with psychological distress following overdose loss, it is essential to examine loss-specific cognitive and emotional factors that shape mental health outcomes. Several theoretically grounded constructs may be particularly relevant. First, continuing bonds refer to the ways in which individuals maintain ongoing connections with the deceased, such as through memories or hallucinations of the deceased or drawing on the deceased as a source of guidance (Field & Filanosky, 2009). While these bonds can be adaptive, they may also reflect difficulty adjusting to the loss in certain contexts. Second, the centrality of the death event captures the extent to which the loss becomes integrated into an individual’s identity and life narrative. Greater centrality has been linked to worse psychological outcomes, as the event comes to define one’s sense of self (Berntsen & Rubin, 2006). Third, meaning-making processes can also impact mental health outcomes through attempts to develop comprehensibility in the death and one’s identity as a result, which demonstrate the extent to which an individual is able to integrate a stressful life event into their overall life narrative (Holland et al., 2010). Such meaning-making processes are central to theories of bereavement and are associated with more adaptive adjustment (Holland et al., 2014). Fourth, unfinished business, including unfulfilled wishes and unresolved conflict, reflects lingering relational concerns that may impede further adaptation to a death and contribute to ongoing distress (Holland et al., 2020).

In addition to these loss-specific processes, broader psychological and interpersonal resources such as resilience and social support may buffer against adverse outcomes. Resilience reflects an individual’s capacity to adapt to stress and has been linked to improved psychological functioning (Connor & Davidson, 2003), while social support plays a critical role in mitigating distress and facilitating recovery (Reime et al., 2024).

### 1.5. Overview of Study

Despite the growing recognition of overdose bereavement as a significant public health concern (Kennedy-Hendricks et al., 2024), there remains a lack of comprehensive research on the mental health challenges faced by individuals who experience overdose loss. The absence of such knowledge limits the ability to identify potential mechanisms for further study that may underlie psychological distress, which in turn can inform the development of targeted, evidence-based interventions. Building on prior work demonstrating the substantial mental health burden associated with overdose bereavement (Bottomley et al., 2022), the present secondary analysis focuses on heterogeneity within the overdose-bereaved population. Specifically, we examine whether continuing bonds, event centrality, loss integration, unfinished business, resilience, and social support are associated with the severity of prolonged grief, depressive, anxiety, and posttraumatic stress symptoms. In doing so, this study moves beyond documenting the prevalence of psychological distress following overdose loss to identifying factors associated with variation in mental health outcomes among overdose-bereaved individuals.

## 2. Materials and Methods

### 2.1. Study Population

The study population included 115 overdose-bereaved individuals who had experienced the death of a loved one or close social network member due to a drug-related overdose. Eligibility was defined as individuals aged 18 years or older who experienced the sudden death of a loved one in the past five years. Participants were recruited via social media (Twitter and Instagram) through a central study page, posts in bereavement support communities when granted permission, and word-of-mouth referrals. Interested participants were directed to a study website that contained detailed information on the study’s purpose, procedures, and research team. Data were collected between August 2019 and September 2021 using Qualtrics online surveys. Upon survey completion, participants received either a $5 Amazon gift card or a charitable donation made on their behalf. All subjects gave their informed consent for inclusion before they participated in the study. The study was conducted in accordance with the Declaration of Helsinki, and the protocol was approved by the University of Memphis Institutional Review Board (PRO-FY2018-685). Further details on the study population and procedures have been previously published by Bottomley et al. (2022). The present study is a secondary analysis of the overdose-bereaved subsample previously reported by Bottomley et al. (2022). Whereas the prior study compared mental health symptom burden across overdose, suicide, and sudden-natural bereavement groups, the present analysis focuses exclusively on overdose-bereaved participants to examine cognitive, emotional, interpersonal, and psychological factors associated with variation in mental health symptom severity.

### 2.2. Outcome Variables

This study has four primary outcomes, which include the symptom severity of prolonged grief disorder (PGD), major depressive disorder (MDD), generalized anxiety disorder (GAD), and post-traumatic stress disorder (PTSD). PGD is characterized by persistent yearning or longing for the deceased as well as intense responses of sorrow to the death of a loved one, reflecting a difficulty adjusting to the loss for more than one year in adults. The Inventory of Complicated Grief (ICG), which uses 19 bereavement-related first-person statements, is a commonly used tool for evaluating indicators of pathological grief and is often used to measure PGD (Mason & Tofthagen, 2019; Prigerson et al., 1995, 1996). Items on the ICG are scored using a five-point Likert scale to measure how often something occurs, ranging from 0 (never) to 4 (always), with higher total scores indicating greater grief symptom severity. The continuous ICG total score was used as the outcome in the primary multivariable analyses. For descriptive purposes only, an ICG score ≥25 was used to identify participants with clinically elevated grief symptoms based on previously established scoring recommendations (Prigerson et al., 1995). Because DSM-5-TR criteria specify that PGD in adults cannot be diagnosed until at least 12 months after the death (American Psychiatric Association, 2022), this descriptive estimate was restricted to participants who were at least 12 months post-loss.

MDD is characterized by distinct episodes lasting at least two weeks, though they often persist much longer, featuring noticeable changes in mood, thinking, and neurovegetative functioning, with periods of remission between episodes (American Psychiatric Association, 2022). The Patient Health Questionnaire-8 (PHQ-8), consisting of 8 items that assess severity of depressive symptoms, is frequently used to measure MDD (Kroenke et al., 2009). Items on the PHQ-8 are scored on a scale of 0 (not at all) to 3 (every day), with a total score ≥10 indicating clinically significant symptoms of MDD.

GAD is characterized by ongoing, excessive worry about multiple areas, such as work or school, that is hard to control, along with physical symptoms like restlessness, fatigue, and difficulty concentrating (American Psychiatric Association, 2022). The Generalized Anxiety Disorder 7-item scale (GAD-7) is a commonly used brief clinical measure of GAD (Spitzer et al., 2006). Items on the GAD-7 scale range from 0 (not at all) to 3 (nearly every day), with a score of ≥10 indicating probable GAD.

Finally, PTSD is characterized by the emergence of specific symptoms after experiencing one or more traumatic events, including intrusive and distressing memories, trauma-related dreams, dissociative reactions such as flashbacks, intense psychological distress when exposed to reminders of the event, and strong physiological responses to related internal or external cues (American Psychiatric Association, 2022). The Posttraumatic Stress Disorder Checklist (PCL-5) is a widely used self-report tool consisting of 20 items for determining PTSD (Blevins et al., 2015). Items on the PCL-5 scale range from 0 (not at all) to 4 (extremely), with composite scores of ≥33 indicating probable PTSD.

### 2.3. Predictor Variables

To better assess the cognitive, emotional, and contextual factors that may be associated with PGD, MDD, GAD, and PTSD symptom severity among overdose-bereaved adults, we examined several predictors that were selected a priori based on theoretical relevance and the prior literature on bereavement and trauma. Predictors include continuing bonds with the deceased, centrality of the death event, meaning making, unfinished business with the deceased, resiliency, and social support. For all measures, higher scores reflect greater levels of the construct (e.g., a higher score on the continuing bonds scale would mean greater continuing bonds after the death).

Continuing bonds with the deceased were measured using 11 items from the Continuing Bonds Scale (CBS), a self-report scale that ranges from 1 (never) to 5 (always) and focuses on internalized, externalized, and legacy statements (Field & Filanosky, 2009). The centrality of the death event was measured using 7 items from the Centrality of Event Scale (CES), a self-report scale that ranges from 1 (strongly disagree) to 5 (strongly agree) and concentrates on turning points in the respondent’s life narrative (Berntsen & Rubin, 2006). Meaning making was measured through 6 items of the Integration of Stressful Life Experiences Scale short form (ISLES-SF), a self-report scale that ranges from 1 (strongly agree) to 5 (strongly disagree) and covers themes of meaning making (i.e., comprehensibility and footing in the world). Comprehensibility evaluates how well a person has been able to understand a stressful experience and adaptively incorporate it into a broader framework for making sense of themselves, others, and the world around them. Footing in the world describes how much the world does or does not feel coherent in the aftermath of a major life stressor (Holland et al., 2010). Items were scored such that higher scores on both ISLES-SF dimensions indicated greater integration of the loss, including greater comprehensibility and greater footing in the world. The experience of unfinished business with a loved one after a loss was measured using a brief 8-item version of the Unfinished Business in Bereavement Scale (UBBS-Brief), a self-report scale that ranges from 1 (not at all distressed) to 5 (extremely distressed) and focuses on declarative statements revolving around persistent relationship incompleteness and, more specifically, lingering regrets and unexpressed feelings through unfulfilled wishes and unresolved conflict (Holland et al., 2020). Resiliency was measured using 2 items from the Connor–Davidson Resilience Scale (CD-RISC), a self-report scale ranging from 1 (not true at all) to 5 (true nearly all of the time) that captures experiences of adaptive coping in the face of adversity (Connor & Davidson, 2003). The perceived availability of social support across various domains was measured using 5 items from the Medical Outcomes Study Social Support Survey (MOS-SS), a self-report survey that ranges from 1 (none of the time) to 5 (all of the time) (Sherbourne & Stewart, 1991).

### 2.4. Covariates

Demographic information included age, gender, race/ethnicity, marital status, education, income, employment status, and geographic location. Other loss-related covariates included the participants’ relationship to the decedent (e.g., parent, child, sibling, etc.), age of decedent, prior psychiatric diagnosis (yes/no), number of previous traumatic deaths, time since loss, and levels of pre-death closeness. Pre-death closeness was measured using the Quality of Relationship Inventory–Bereavement version (QRI-B), a tool developed by members of our team to measure how individuals perceive their relationships, focusing on aspects such as conflict and the depth or closeness of a specific relationship prior to the person’s death (Bottomley & Neimeyer, 2022).

### 2.5. Statistical Analysis

Descriptive statistics were calculated for all study variables, and distributions were examined to assess normality and identify potential outliers. Pearson correlation coefficients were used to evaluate bivariate relationships among study variables.

Four separate multivariable linear regression models were constructed to estimate independent associations between predictor variables and each continuous mental health outcome (severity of PGD, MDD, GAD, and PTSD symptoms). Eight theoretically selected bereavement-related constructs central to the study aims were included as primary predictors: continuing bonds, centrality of the death event, two dimensions of integration of the loss (footing in the world and comprehensibility), two dimensions of unfinished business (unfulfilled wishes and unresolved conflict), resilience, and social support. Consistent with the exploratory aims of the study and modest sample size, analyses prioritized model parsimony to reduce the risk of overfitting. All predictors were modeled as continuous variables. Internal consistency was assessed using Cronbach’s alpha for multi-item scales and subscales and the Spearman–Brown coefficient for the two-item CD-RISC. Descriptive statistics and internal consistency estimates for all study measures are presented.

Given the modest sample size, model complexity was limited by restricting covariate adjustment to time since loss, age, and pre-death psychiatric illness based on their theoretical relevance and potential to confound associations between the primary predictors and mental health outcomes. Gender, pre-death closeness, and relationship to the decedent (operationalized as parent bereavement versus all other relationships) were considered as potential covariates but were not retained in final models to preserve model parsimony; none of these variables meaningfully altered the estimated associations of interest in sensitivity analyses. Missing data were handled using listwise deletion, and final analytic sample size was *n* = 112 for each regression model.

Regression diagnostics were evaluated for each model. Linearity and homoscedasticity were evaluated by visual inspection of residual-versus-fitted plots, and normality of residuals was assessed using Q-Q plots. Residual-versus-fitted plots suggested mild heteroscedasticity; therefore, heteroscedasticity-robust standard errors were used for all regression models. Potentially influential observations were assessed using leverage-versus-residual-squared plots. Multicollinearity among predictors was assessed using variance inflation factors (VIF), with values above conventional thresholds (VIF > 3) indicating potential concern (Kim, 2019). Model diagnostics indicated no substantial multicollinearity or influential observations that warranted exclusion. Unstandardized regression coefficients (*B*) and corresponding 95% confidence intervals (CIs) were reported. Given the exploratory nature of the study and the number of associations examined, no formal adjustment for multiple comparisons was applied. Findings were interpreted based on the pattern and direction of associations, effect estimates, and 95% confidence intervals, and should be considered hypothesis-generating. Statistical tests were two-sided, with *p* < 0.05 considered statistically significant. In regression tables, statistical significance was denoted as *p* < 0.001, *p* < 0.01, and *p* < 0.05. All analyses were conducted using Stata version 19.5 (StataCorp LLC, College Station, TX, USA).

## 3. Results

### 3.1. Descriptive Characteristics

Of 115 participants, 85.2% were female, 86.1% were White, and 60.0% were married (Table 1). The mean age of participants was 50 years, while the mean age of decedents was 33 years. All study participants (100%) completed high school, and 53.1% had a college or post-graduate degree. For 64.4% of participants, the decedent was their child, and for 16.5% the decedent was their sibling. Prior to the loss, 20.9% of participants reported being diagnosed with a psychiatric disorder. Participants experienced 1.3 previous traumatic deaths on average, including the index death. The average time since loss was 21.8 months. Additional demographic information is provided in Table 1.

### 3.2. Descriptive Statistics and Internal Consistency

Means, standard deviations, and internal consistency estimates for the study measures are presented in Table 2. In the current sample, internal consistency was strong for all outcome measures (α = 0.87–0.91). Internal consistency was acceptable to excellent for most predictor variables (αs = 0.75–0.89), with the exception of the UBBS-Unresolved Conflict subscale (α = 0.60). The two-item CD-RISC demonstrated acceptable reliability (Spearman–Brown = 0.70).

### 3.3. Bivariate Associations

Pearson correlation coefficients among study variables are presented in Table 3. Overall, the four mental health outcomes were moderately to strongly correlated with one another (r = 0.44–0.71, all *p* < 0.001), indicating substantial overlap in symptomatology. Several bereavement-related processes were associated with higher mental health outcome symptom severity at the bivariate level. Greater centrality of the death event was positively correlated with symptom severity of all four mental health outcomes, while greater continuing bonds with the deceased were associated with higher PGD symptoms but no other outcomes. In contrast, greater meaning making, particularly the ability to make sense of the loss (i.e., comprehensibility), was negatively associated with all four mental health outcomes. Indicators of unfinished business, particularly unfulfilled wishes, were positively correlated with all mental health outcomes. Greater resilience was negatively correlated with MDD, GAD, and PTSD symptoms, while greater social support was negatively correlated with MDD and GAD symptoms. Among covariates, time since loss and age were negatively associated with several outcomes, while psychiatric history was modestly associated with depressive symptoms. Overall, correlations among predictor variables were small to moderate in magnitude, supporting their inclusion as distinct constructs in multivariable models.

### 3.4. Multivariable Regression Analyses

Results from multivariable linear regression models examining associations between bereavement-related factors and mental health outcomes are presented in Table 4.

#### 3.4.1. Prolonged Grief Disorder

The average grief score on the ICG was 35.4 (standard deviation = 11.7). Among the 79 participants who were at least 12 months post-loss, 61 (77.2%) had an ICG score ≥25, indicating clinically elevated grief symptoms. In the multivariable model, greater continuing bonds (*B* = 0.36, 95% CI [0.14, 0.59], *p* < 0.01) and higher centrality of the death event (*B* = 0.73, 95% CI [0.26, 1.21], *p* < 0.01) were associated with increased PGD symptom severity. Greater comprehensibility was associated with lower PGD symptoms (*B* = −1.04, 95% CI [−1.79, −0.29], *p* < 0.01).

#### 3.4.2. Major Depressive Disorder

The average score on the PHQ-8 was 10.4 (standard deviation = 6.0), with 59 participants (51.3%) screening positive for probable MDD. Greater centrality of the death event (*B* = 0.22, 95% CI [0.04, 0.39], *p* < 0.05) and unfulfilled wishes (*B* = 0.28, 95% CI [0.08, 0.48], *p* < 0.01) were associated with higher depressive symptoms. In contrast, greater integration of the loss through finding footing in the world (*B* = −0.77, 95% CI [−1.11, −0.43], *p* < 0.001), higher resilience (*B* = −0.53, 95% CI [−1.02, −0.03], *p* < 0.05), and greater social support (*B* = −0.22, 95% CI [−0.42, −0.02], *p* < 0.05) were associated with lower depressive symptoms. Additionally, more time since the loss was significantly associated with lower depressive symptoms (*B* = −0.08, 95% CI [−0.14, −0.03], *p* < 0.01).

#### 3.4.3. Generalized Anxiety Disorder

The average score on the GAD-7 was 7.9 (standard deviation = 5.6), with 36 participants (31.3%) screening positive for probable GAD. In the model for anxiety symptoms, greater continuing bonds (*B* = 0.12, 95% CI [0.01, 0.23], *p* < 0.05) and higher centrality of the death event (*B* = 0.29, 95% CI [0.09, 0.49], *p* < 0.01) were associated with increased anxiety symptoms. Higher resilience (*B* = −0.82, 95% CI [−1.47, −0.17], *p* < 0.05) and older age (*B* = −0.18, 95% CI [−0.28, −0.08], *p* < 0.001) were associated with lower anxiety symptoms.

#### 3.4.4. Posttraumatic Stress Disorder

The average score on the PCL-5 was 33.3 (standard deviation = 14.7), with 53 participants (46.1%) screening positive for probable PTSD. For PTSD symptoms, greater unfulfilled wishes (*B* = 0.80, 95% CI [0.22, 1.39], *p* < 0.01) was associated with increased symptom severity. In contrast, greater integration of the loss through finding footing in the world (*B* = −1.05, 95% CI [−2.08, −0.02], *p* < 0.05) and higher resilience (*B* = −2.01, 95% CI [−3.53, −0.49], *p* = 0.01) were associated with lower PTSD symptoms.

## 4. Discussion

### 4.1. Summary

This study examined the association of multiple cognitive, emotional, and interpersonal factors with mental health outcomes among individuals bereaved by drug overdose. Overall, findings underscore that bereavement following overdose is a complex and multidimensional process characterized by both loss-specific cognitive processes and broader psychological resources. Several consistent patterns emerged across outcomes, alongside important differences that suggest distinct patterns may characterize prolonged grief, depression, anxiety, and posttraumatic stress within this population.

The variation in factors associated with mental health outcomes supports a multidimensional view of bereavement-related psychopathology, particularly among those who experience the fatal overdose of a close other. Prolonged grief symptoms were associated with event centrality, continuing bonds, and comprehensibility. In contrast, depressive symptoms were associated with a broader constellation of factors, including event centrality, finding footing in the world, resilience, social support, and unfulfilled wishes. Anxiety symptoms were associated with event centrality, continuing bonds, and resilience, while PTSD symptoms were associated with unfulfilled wishes, finding footing in the world, and resilience. These distinctions suggest that different factors may be relevant to different forms of distress following overdose loss. This has important implications for both theoretical models of bereavement after fatal overdose and future intervention development.

### 4.2. Integration with the Existing Literature

Our results underscore the relevance of loss-related cognitive processes in shaping adverse mental health outcomes. Greater centrality of the death event was consistently associated with higher symptom severity for prolonged grief, depression, and anxiety, aligning with prior research demonstrating that when a loss becomes a defining aspect of one’s identity, it is more likely to contribute to persistent distress and maladaptive coping (Brookman et al., 2024). Similarly, continuing bonds were associated with higher symptom severity for both PGD and GAD, suggesting that in the context of overdose loss (often characterized by suddenness, stigma, and perceived preventability), ongoing attachment may reflect difficulty adapting to the loss. The findings also highlight the importance of unfinished business, particularly unfulfilled wishes, which were associated with increased depression and PTSD symptoms. This aligns with conceptualizations of grief that emphasize unresolved relational or existential concerns as central drivers of distress (Smid, 2020). In overdose loss, where individuals may experience regret or perceived missed opportunities due to the often sudden nature of the death, these processes may be particularly salient.

In contrast, greater meaning making was associated with lower symptom severity. Importantly, the distinction between comprehensibility and footing in the world appeared meaningful in this sample, suggesting that different aspects of meaning making may operate differently across outcomes. Specifically, greater footing in the world was associated with lower MDD and PTSD symptoms, whereas greater comprehensibility was associated with lower PGD symptoms. These findings are consistent with broader theories of meaning making in the bereavement literature that identify meaning reconstruction as an important component of adaptive adjustment (Smid, 2020). Greater resilience was also associated with lower depression, anxiety, and PTSD symptoms. This pattern is consistent with prior research examining resilience among bereaved parents (Cheng et al., 2026; Rasouli et al., 2023; Vegsund et al., 2019). Finally, greater social support was associated with lower depression symptom severity, consistent with prior research demonstrating the buffering role of support systems in mitigating psychological distress following traumatic loss (Calhoun et al., 2022). Given the cross-sectional design, however, the directionality of these associations cannot be established.

This study also contributes to a growing body of literature challenging the assumption that time alone leads to recovery following bereavement. Prior research among individuals bereaved by drug-related deaths has demonstrated that symptom severity does not necessarily decrease linearly with time and that individuals one to two years post-loss may exhibit particularly elevated levels of prolonged grief symptoms (Titlestad & Dyregrov, 2024). Consistent with this work, time since loss was associated with only one of four mental health outcomes in the current study, whereas several loss-specific cognitive, emotional, and relational processes such as centrality of the death event, meaning-making, continuing bonds, and unfinished business were associated with multiple outcomes. These findings suggest that psychological adjustment following overdose bereavement may be related not only to the amount of time elapsed since the death, but also to how individuals process and adapt to the loss.

### 4.3. Implications and Future Research Directions

Although the cross-sectional design does not establish that modifying the factors examined in this study would improve mental health outcomes, the observed associations identify several processes that may warrant investigation in future intervention research. First, the associations involving meaning-making and event centrality suggest that future intervention studies could examine whether approaches designed to facilitate cognitive integration and sensemaking following overdose loss improve mental health outcomes. Meaning-centered therapy (Lichtenthal et al., 2019) and narrative-based interventions (Babar et al., 2024) provide examples of approaches that could be evaluated specifically among overdose-bereaved populations.

Similarly, the associations between greater resilience and lower MDD, GAD, and PTSD symptom severity suggest that future research could test whether interventions designed to strengthen coping capacity, emotional regulation, and psychological flexibility improve outcomes among overdose-bereaved individuals. The prior literature provides a rationale for examining resilience-promoting approaches in bereavement contexts and demonstrates the important role that healthcare professionals play in fostering resilience among bereaved parents (Cheng et al., 2026; Rasouli et al., 2023). Holistic and empowering approaches that address the multidimensional processes of resilience should also be studied in the context of overdose bereavement, as these approaches warrant evaluation for potential benefits across multiple domains of mental health (Boelen et al., 2006; Stroebe & Schut, 2010).

The associations between greater social support and lower depressive symptom severity also suggest that interventions designed to strengthen support networks warrant further investigation among overdose loss survivors. Given the stigma often associated with overdose deaths, individuals may face barriers to seeking or receiving support (Dyregrov & Selseng, 2022). A study of online suicide loss support groups demonstrated how they significantly help individuals cope with sudden and particularly violent deaths by allowing them to seek and share support as well as find and share resources against the backdrop of an often stigmatized death (Lestienne et al., 2021). Therefore, enhancing both formal and informal support networks, including peer-based and online grief support communities, may be particularly important for this population.

Additionally, the associations involving unfinished business and continuing bonds suggest that future intervention research could examine approaches addressing unresolved relational concerns and ongoing connections with the deceased. Techniques such as imaginal dialogue (Wetherell, 2012), expressive writing (Yao et al., 2025), and other forms of symbolic communication and connection represent potential approaches for empirical evaluation in overdose-bereaved populations.

Future research should build on these findings using longitudinal designs to examine the temporal relationships between these factors and bereavement trajectories over time. Of particular interest is whether and how potentially modifiable variables such as continuing bonds and unfinished business are prospectively associated with subsequent mental health and adaptive outcomes. Additionally, larger studies should explore other contextual factors that may uniquely shape the experience of overdose loss. Well-powered intervention studies are needed to test whether targeting meaning-making, unfinished business, continuing bonds, and resilience can effectively reduce psychological distress in this population. Future studies with larger and more diverse samples should also examine potential heterogeneity in bereavement experiences and associated mental health outcomes across gender and relationship-to-decedent subgroups. Integrating quantitative and qualitative approaches would also provide a more comprehensive understanding of the lived experience of overdose bereavement.

Future research should also examine the role of perceived intentionality in overdose bereavement and examine whether bereavement experiences and mental health outcomes differ according to perceptions of intent. In particular, studies directly comparing bereavement following unintentional overdose, intentional drug-related death, and suicide may help clarify similarities and differences in the cognitive, emotional, and interpersonal processes associated with mental health outcomes.

## 5. Limitations

Several limitations should be considered when interpreting these findings. First, the cross-sectional design precludes causal inference; it is not possible to determine the directionality of associations between predictors and mental health outcomes. Second, the modest sample size limits the ability to examine more complex models or include additional covariates without risking overfitting. Although efforts were made to prioritize theoretically relevant and empirically supported covariates, it is possible that unmeasured confounding remains. Third, data were collected via self-report measures, which may be subject to recall bias or social desirability effects. Internal consistency was lower for the UBBS unresolved-conflict subscale (α = 0.60), and findings involving this construct should therefore be interpreted cautiously. Additionally, recruitment through online platforms, including online grief support groups, limits generalizability. Individuals engaged in such groups may differ systematically from those not receiving or seeking support online, for example, by having greater access to resources, higher help-seeking behavior, or differing levels of distress. As a result, our estimates may be biased and may not fully reflect the experiences of overdose loss survivors who are not connected to support networks. The sample was also predominantly female and White and included a substantial proportion of participants who had lost a child, which may further limit generalizability. Bereavement experiences and mental health outcomes may vary by gender, race/ethnicity, and relationship to the deceased, and the associations observed in this study may therefore partly reflect the composition of the sample. Although sensitivity analyses indicated that adjustment for gender, pre-death closeness, and relationship-to-decedent did not materially alter the primary associations, larger and more diverse samples are needed to examine whether these associations vary across demographic and relationship groups. The survey also did not distinguish between overdose deaths perceived by respondents as intentional versus unintentional. Perceived intentionality may influence the bereavement experience and could contribute to heterogeneity in psychological responses following overdose loss.

## 6. Conclusions

In summary, this study highlights the complex and multifaceted nature of bereavement following overdose loss. Findings demonstrate that distinct cognitive, emotional, and interpersonal processes are associated with the severity of different mental health outcomes. By identifying factors associated with symptom severity across multiple domains, this study contributes to a growing but limited literature on overdose bereavement and identifies potentially modifiable processes that warrant further longitudinal and intervention research.

## Figures and Tables

**Table 1:** Demographics and Loss-Related Covariates Among Overdose-Bereaved Adults.

Features		*n* (115)	%
**Gender**	Male	17	14.78
	Female	98	85.22
**Race/Ethnicity**	White	99	86.09
	Black	5	4.35
	Hispanic	4	3.48
	Other	7	6.09
**Marital Status**	Married	69	60.0
	Single	17	14.78
	Divorced or Widowed	29	25.22
**Education**	High School Graduate	13	11.30
	Some College/Trade/Technical/Vocational	41	35.65
	College Graduate	34	29.57
	Post Graduate Degree	27	23.48
**Income (*n* = 112)**	Under $30,000	8	7.14
	$30,000–$74,999	46	41.07
	$75,000+	58	51.79
**Work**	Employed Full Time	57	49.57
	Employed Part Time	9	7.83
	Not Working	18	15.65
	Student	7	6.09
	Retired	24	20.87
**Geographic Location (*n* = 114)**	Rural	11	9.65
	Small Town	18	15.79
	Small City	22	19.30
	Large City	19	16.67
	Suburb	44	38.60
**Relationship to Decedent**	Decedent was the Child	74	64.35
	Sibling	19	16.52
	Parent	2	1.74
	Spouse/Partner	8	6.96
	Grandparent	2	1.74
	Friend	6	5.22
	Other	4	3.48
**Pre-loss Psychiatric Disorder Diagnosis**	Yes	24	20.87
	No	91	79.13
		Mean	St. Deviation
**Age**		50.3	13.9
**Age of Decedent**		32.6	9.7
**Number of Previous Traumatic Deaths**		1.3	1.8
**Time Since Loss (Months)**		21.8	15.3
**Degree of Pre-Death Closeness**		25	5.1
**Degree of Pre-Death Conflict**		10.7	3.9

Data are presented as frequencies and percentages or means and standard deviations, as appropriate. Percentages were calculated using nonmissing observations; variable-specific denominators are reported for variables with missing data.

**Table 2:** Descriptive Statistics and Internal Consistency of Study Measures.

Measure	Instrument	Possible Score Range	Mean	SD	Reliability Coefficient
**Mental health outcomes**					
Prolonged grief symptoms	ICG	0–76	35.4	11.7	0.87
Depressive symptoms	PHQ-8	0–24	10.4	6.0	0.89
Generalized anxiety symptoms	GAD-7	0–21	7.9	5.6	0.91
Posttraumatic stress symptoms	PCL-5	0–80	33.3	14.7	0.90
**Bereavement-related predictors**					
Continuing bonds	CBS	11–55	41.6	8.5	0.85
Centrality of the death event	CES	7–35	30.5	4.8	0.89
Integration: comprehensibility	ISLES-SF	3–15	7.4	3.4	0.83
Integration: footing in the world	ISLES-SF	3–15	8.9	3.2	0.88
Unfinished business: unfulfilled wishes	UBBS-Brief	4–20	10.8	4.6	0.75
Unfinished business: unresolved conflict	UBBS-Brief	4–20	8.3	3.5	0.60
Resilience	CD-RISC	2–10	6.9	1.9	0.70
Social Support	MOS-SS	5–25	18.0	4.5	0.85

Data are presented as ranges, means, standard deviations, and Spearman–Brown coefficients (for the CD-RISC) and Cronbach’s α (all other instruments).

**Table 3:** Bivariate Correlations Among Bereavement-Related Factors and Mental Health Outcomes.

	1	2	3	4	5	6	7	8	9	10	11	12	13	14	15	16	17
**1. PGD Severity**	1.00																
**2. MDD Severity**	0.55 ***	1.00															
**3. GAD Severity**	0.44 ***	0.71 ***	1.00														
**4. PTSD Severity**	0.52 ***	0.63 ***	0.57 ***	1.00													
**5. Continuing Bonds**	0.34 ***	0.10	0.10	0.08	1.00												
**6. Centrality of Event**	0.43 ***	0.33 ***	0.25 **	0.27 **	0.25 **	1.00											
**7. Comprehensibility**	−0.34 ***	−0.48 ***	−0.30 **	−0.30 ***	−0.07	−0.34 ***	1.00										
**8. Footing in World**	−0.38 ***	−0.18	−0.16	−0.14	−0.12	−0.28 **	0.52 ***	1.00									
**9. Unfulfilled Wishes**	0.23 *	0.34 ***	0.30 **	0.41 ***	0.14	0.00	0.01	0.00	1.00								
**10. Unresolved Conflict**	0.15	0.13	0.16	0.29 **	0.14	0.02	−0.09	−0.11	0.43 ***	1.00							
**11. Resilience**	−0.17	−0.42 ***	−0.39 ***	−0.42 ***	0.26 **	−0.21 *	0.35 ***	0.22 *	−0.07	−0.15	1.00						
**12. Social Support**	−0.15	−0.33 ***	−0.26 **	−0.16	0.07	0.13	0.21 *	0.05	−0.29 **	−0.15	0.22 *	1.00					
**13. Time Since Loss**	−0.17	−0.31 ***	−0.22 *	−0.27 **	0.08	−0.05	0.04	−0.04	−0.15	−0.16	0.20 *	0.04	1.00				
**14. Pre−death Closeness**	0.23 *	0.07	0.00	0.15	0.32 ***	0.25 **	−0.10	−0.27 **	−0.03	−0.16	−0.17	0.30 **	0.07	1.00			
**15. Age**	−0.00	−0.10	−0.33 ***	−0.19 *	0.12	0.34 ***	−0.13	−0.14	−0.36 ***	−0.09	−0.02	0.17	0.10	0.03	1.00		
**16. Gender**	−0.02	0.12	0.07	0.06	−0.06	0.16	−0.19 *	−0.04	−0.08	−0.20 *	0.10	0.15	0.03	−0.03	0.11	1.00	
**17. Psychiatric History**	−0.06	−0.21 *	−0.11	−0.16	−0.01	0.14	−0.09	−0.14	−0.29 **	−0.18	0.06	0.14	0.11	−0.05	0.41 ***	−0.03	1.00

Note: * *p* < 0.05, ** *p* < 0.01, *** *p* < 0.001. Values represent bivariate correlation coefficients among study measures.

**Table 4:** Multivariable Linear Regression Models of Factors Associated with Mental Health Symptom Severity.

	PGD *B* (95% CI)	MDD *B* (95% CI)	GAD *B* (95% CI)	PTSD *B* (95% CI)
**Continuing Bonds**	0.36 (0.14, 0.59) **	0.09 (−0.02, 0.20)	0.12 (0.01, 0.23) *	0.19 (−0.10, 0.48)
**Centrality of Event**	0.73 (0.26, 1.21) **	0.22 (0.04, 0.39) *	0.29 (0.09, 0.49) **	0.44 (−0.39, 1.27)
**Footing in the World**	−0.41 (−1.13, 0.31)	−0.77 (−1.11, −0.43) ***	−0.23 (−0.59, 0.13)	−1.05 (−2.08, −0.02) *
**Comprehensibility**	−1.04 (−1.79, −0.29) **	0.09 (−0.16, 0.34)	−0.00 (−0.29, 0.29)	0.11 (−0.48, 0.70)
**Unfulfilled Wishes**	0.11 (−0.36, 0.59)	0.28 (0.08, 0.48) **	0.09 (−0.13, 0.32)	0.80 (0.22, 1.39) **
**Unresolved Conflict**	−0.06 (−0.59, 0.48)	−0.23 (−0.49, 0.02)	−0.04 (−0.29, 0.21)	0.28 (−0.40, 0.95)
**Resilience**	0.27 (−1.11, 1.64)	−0.53 (−1.02, −0.03) *	−0.82 (−1.47, −0.17) *	−2.01 (−3.53, −0.49) *
**Social Support**	−0.38 (−0.85, 0.09)	−0.22 (−0.42, −0.02) *	−0.17 (−0.36, 0.03)	0.11 (−0.45, 0.66)
**Time Since Loss**	−0.11 (−0.22, 0.00)	−0.08 (−0.14, −0.03) **	−0.05 (−0.10, 0.01)	−0.13 (−0.28, 0.02)
**Age**	−0.10 (−0.24, 0.04)	−0.02 (−0.10, 0.06)	−0.18 (−0.28, −0.08) ***	−0.19 (−0.41, 0.02)
**Psychiatric History**	0.06 (−4.53, 4.64)	−1.72 (−4.29, 0.85)	1.17 (−1.19, 3.52)	1.03 (−6.16, 8.22)

Note: * *p* < 0.05, ** *p* < 0.01, *** *p* < 0.001. Values represent unstandardized regression coefficients (*B*) with 95% confidence intervals. All models adjusted for age, time since loss, and pre-death psychiatric illness. *N* = 112 for each model.

## Data Availability

The data supporting the conclusions of this article will be made available by the authors on request. The data are not publicly available due to ethical restrictions and the need to protect the privacy and confidentiality of the research participants.
